# The effectiveness of multi-component interventions targeting physical activity or sedentary behaviour amongst office workers: a three-arm cluster randomised controlled trial

**DOI:** 10.1186/s12889-020-09433-7

**Published:** 2020-09-01

**Authors:** Carla F. J. Nooijen, Victoria Blom, Örjan Ekblom, Emerald G. Heiland, Lisa-Marie Larisch, Emil Bojsen-Møller, Maria M. Ekblom, Lena V. Kallings

**Affiliations:** 1 The Swedish School of Sport and Health Sciences (GIH), Box 5626, SE-114 86 Stockholm, Sweden; 2 The Department of Public Health Sciences, Karolinska Institutet, Stockholm, Sweden; 3 The Department of Neuroscience, Karolinska Institutet, Stockholm, Sweden; 4 Department of Public Health and Caring Sciences, Family Medicine, Uppsala University, Uppsala, Sweden

**Keywords:** Workplace, Office workers, Sedentary behaviour, Physical activity, Intervention, Device-based, Self-report

## Abstract

**Background:**

Interventions to increase physical activity or reduce sedentary behaviour within the workplace setting have shown mixed effects. This cluster randomised controlled trial assessed whether multi-component interventions, focusing on changes at the individual, environmental, and organisational levels, either increased physical activity or reduced sedentary behaviour, compared to a passive control group.

**Methods:**

Teams of office-workers from two companies participated in one of two interventions (iPA: targeting physical activity; or iSED: targeting sedentary behaviour), or wait-list control group (C). Exclusion criterion was very high physical activity level (MVPA ≥30 min/day in ≥10 min bouts every day). Randomisation occurred at the level of workplace cluster, and groups were randomly allocated (1:1) with stratification for company and cluster size. Personnel involved in data collection and processing were blinded to group allocation. Both interventions included five sessions of cognitive behavioural therapy counselling for 6 months. iPA included counselling focused on physical activity, access to a gym, and encouragement to exercise, and go for lunch walks. iSED included counselling on sedentary behaviour and encouragement to reduce sitting and increase engagement in standing- and walking-meetings. At baseline and the 6-month mark accelerometers were worn on the hip and thigh for 7 days. The primary outcomes were group differences in time spent in moderate-to-vigorous intensity physical activity (%MVPA) and in sedentary behaviour (%), analysed using Bayesian multilevel modelling for those with complete data.

**Results:**

Two-hundred and sixty three office workers (73% women, mean age 42 ± 9 years, education 15 ± 2 years) were randomised into 23 cluster teams (iPA *n* = 84, 8 clusters; iSED *n* = 87, 7 clusters; C *n* = 92, 7 clusters). No significant group differences (posterior mean ratios: 95% credible interval) were found after the intervention for %MVPA or for %Sedentary. %MVPA: iPA vs C (0·04: − 0·80–0·82); iSED vs C (0·47: − 0·41–1·32); iPA vs iSED (0·43: − 0·42–1·27). %Sedentary: iPA vs C (1·16: − 1·66–4·02); iSED vs C (− 0·44: − 3·50–2·64); iPA vs iSED (− 1·60: − 4·72–1·47).

**Conclusions:**

The multi-component interventions focusing on either physical activity or sedentary behaviour were unsuccessful at increasing device-measured physical activity or reducing sedentary behaviour compared to a control group.

**Trial registration:**

ISRCTN, ISRCTN92968402. Registered 27/2/2018, recruitment started 15/03/2018,

## Background

Physically inactive and sedentary lifestyles are acknowledged public health problems [1]. In Sweden, adults spend about two-thirds of their daily time sedentary and the vast majority do not meet the national physical activity recommendations [2]. A substantial proportion of wake time is spent at work and the nature of an office job is likely to increase daily sedentary time [3]. Even though most offices in Sweden have sit-stand desks, office workers spend 60% of their time sedentary, 28% standing, and only 12% walking [4].

When comparing the effects of physical activity and sedentary behaviour on health, previous studies suggest that increasing physical activity might have more impact on reducing the risk for chronic conditions and mortality compared to decreasing sedentary behaviour [5]. However, when it comes to the effects of physical activity and sedentary behaviour on mental health and cognition it is unknown whether one of these behaviours might have more of an impact than the other [6, 7].

Previous interventions aspiring to increase physical activity or reduce sedentary behaviour have shown mixed effects [8, 9]. The importance of focusing the intervention on only one behaviour, sedentary behaviour or physical activity, has been stressed [8, 9]. Furthermore, behavioural change interventions seem to require behaviour specific and multilevel ecological models, as health behaviours are influenced at multiple and interacting levels, as well as by the environmental context [10, 11]. Another aspect to consider are the attitudes towards interventions [12], which might differ and thereby influence the effectiveness of an intervention but also the success of later implementation [13]. For example, going to the gym may for some be a more challenging endeavour than using a sit-stand desk at work. Also cost-effectiveness needs to be considered since costs of interventions may differ, e.g. comparing the costs of a gym card and a sit-stand desk [14]. Studies comparing different interventions are thus warranted.

Besides the importance of designing interventions based on theory, incorporating preferences of the population that will receive the intervention might be critical [15]. According to a survey answered by more than 500 Swedish office workers the most commonly reported perceived barrier for standing or walking was that sitting was a habit [4]. As cognitive behavioural therapy (CBT) is a widely used method for changing habits, we chose to use CBT as the basis of our interventions in addition to motivational interviewing techniques [16]. In the same study it was found that preferred strategies for breaking up sedentary behaviour were standing or walking meetings [4]. Therefore, we designed our interventions accordingly. Incorporating these group-based intervention strategies in an office environment requires a cluster randomised design to minimise contamination between groups [17].

As described in the published protocol [18], we performed a 3-armed cluster randomised controlled trial (RCT) to assess the effectiveness of multi-component interventions to increase physical activity or reduce sedentary behaviour, among office workers, in order to improve mental health and cognition.

## Methods

The study was a 6-month cluster RCT with 3-arms of which one was a wait-list control group. The objective of the current paper was to assess whether the multi-component interventions, incorporating individual, environmental, and organisational changes, increased physical activity or reduced sedentary behaviour after the 6-month interventions. Effects of the two interventions, either targeting physical activity (iPA) or sedentary behaviour (iSED), were compared to a wait-list control group and to each other. We hypothesised that participating in the iPA intervention would increase time spent in moderate-to-vigorous intensity physical activity (MVPA), and participating in the iSED intervention would decrease time spent sedentary.

Ethical approval was granted by The Stockholm regional ethical review board (2017/2409–31/1). All participants provided written informed consent before the first data collection. The study was conducted in accordance with the CONSORT guidelines for cluster RCTs. The trial was prospectively registered as ISRCTN92968402 on 27/02/2018, recruitment started 15/03/2018 (10.1186/ISRCTN92968402). A study protocol [18], including a detailed description and rationale of the trial, has been published earlier, the most important details are provided below.

### Study population

From two Swedish product and service producing companies (with 3 different office locations), 2033 office workers were invited to participate in the study. The target was to include 330 persons, based on sample size calculations [18]. Inclusion criteria were ages 18–70 years and able to stand and exercise. Persons were excluded if they were very physically active, according to device-measured MVPA of more than 30 min/day in prolonged bouts (≥10 min) every day. Sedentary behaviour was not used as an exclusion criterion, as a previous cross-sectional study in a comparable sample showed that nearly all office workers reported high levels of sedentary behaviour [4].

### Randomisation and masking

Clusters consisted of teams of office workers grouped by Human Resources (HR) personnel at each company. Minimum amount of persons per cluster was five and other considerations taken into account were: 1) having a team or line manager, 2) having regular group meetings, and 3) having limited regular meetings with other teams. Randomisation into groups was performed after finishing baseline data collection and was done on a cluster level [18]. Groups were randomly allocated (1:1) with stratification for company and cluster size (large vs. small). Matched randomisation was applied to realise logistical capacity. The random allocation sequence was setup using a computer-generated random number list, and randomisation was performed by an independent researcher not involved in data collection. All participants were informed on allocation after baseline data collection via e-mail sent from another independent researcher not involved in data collection. Personnel involved in data collection and processing were blinded for group allocation.

### Interventions

Two multi-component interventions aiming to either increase physical activity or reduce sedentary behaviour were designed based on the ecological framework addressing multiple levels including individual, environmental, and organisational [18, 19]. Both interventions lasted 6 months and focused on work time as well as leisure time, to decrease the risk that participants compensate by e.g. decreasing sedentary behaviour at work but at the same time increasing sedentary behaviour during leisure time [20].

The individual support included motivational counselling that had a comparable design but a different focus in the two interventions. This counselling was provided by professional health coaches from a health promotion company who received additional training on CBT and on physical activity and sedentary behaviour. There were five sessions; three individual (45–60 min) and two group sessions (90 min) spread out during the 6-months intervention period. The content of the counselling sessions was standardised using manuals with checklists.

A team leader was appointed to each cluster to deliver the environmental and organisational components of the interventions, and to encourage all participants to remain in the study. HR personnel of each company asked one participant in each cluster to be the team leader, for which no formal requirements were set, but judgement of HR personnel on a suitable individual was adopted. At the start of the intervention, one of the researchers contacted all team leaders by phone to coordinate the logistics of implementing the different components of the assigned intervention.

#### Intervention to promote physical activity level (iPA)

The intervention aiming to promote MVPA included:

- Individual: motivational counselling using CBT towards increasing time spent in MVPA, including individual feedback on physical activity (MVPA and steps/day).
- Environmental: access to a commercial gym (6 months), exercise sessions and lunch walks organised by team leaders, and the provision of company bikes.
- Organisational: team leaders encouraged employees to be physically active inside and outside of working hours, including commuting to work.

#### Intervention to reduce sedentary behaviour (iSED)

The intervention aiming to reduce sedentary behaviour, including breaking up prolonged sitting, included:

- Individual: motivational counselling using CBT towards reducing time in sedentary behaviour and breaking up prolonged sitting, including individual feedback on sedentary behaviour patterns (sedentary behaviour and steps/day).
- Environmental: team leaders were instructed to organise standing and walking meetings. Note that companies already provided their employees with sit-stand desks [4].
- Organisational: team leaders encouraged employees to reduce sedentary behaviour during work, in meetings, and while sitting behind their desks as well as outside of working hours including lunch and commuting.

#### Control group (C)

A passive wait-list group, which received one of the described interventions after the follow-up measurement (at 6 months).

### Data collection

Inclusion of participants started March 2018 and ended November 2018, after all employees at the companies had been informed about the study. Data collection of the RCT finished in May 2019. At both baseline and the 6-month follow-up, all participants filled out online surveys and participated in device-measured physical activity and sedentary behaviour measurements.

### Participants’ characteristics

Characteristics of participants were collected from the online survey, and included: company of employment, age, gender, years of education, family status (married/living together: yes vs. no), working fulltime (yes vs. no), and smoking (yes vs. no). Weight and height measured at baseline were used to calculate body mass index (BMI; kg/m^2^).

### Outcome variables

#### Device-measured physical activity and sedentary behaviour

Participants were instructed to wear an Actigraph GT3X accelerometer (Actigraph GT3X, Fort Walton Beach, Florida, USA) on the hip during wake time and on the wrist during in-bed time, along with an ActivPal inclinometer on the frontal aspect of the mid-thigh (*activPAL*™ 3 activity monitors, PAL technologies limited, Glasgow, UK) during 7 days. During the measurement period, participants noted in-bed times, working hours, sleep quality, and daytime sleepiness in a diary.

##### *Actigraph*

Accelerations from 3 axes (vector magnitude, VM) were sampled at a frequency of 30 Hz. Minimum requirement for data inclusion was 600 min of valid wear time during waking hours, based on diary information, on at least 4 days. Non-wear time was defined as at least 60 consecutive minutes with no movement (VM = 0 counts per minute, cpm), with an allowance of maximum 2 min of activity. The primary outcome was time spent in MVPA (> 2690 cpm), expressed as a percentage of wear time based on an average of all available days. Secondary outcome measures (also expressed as percentage of wear time) were light (200–2689 cpm), moderate- (2690–6166 cpm), and vigorous-intensity physical activity (> 6167 cpm) as averages over available days, as well as weekday only averages [21].

##### *ActivPAL*

Inclination of the thigh was obtained to quantify time spent sedentary, standing, and walking. Sedentary was defined as the thigh in a horizontal position, i.e. sitting or lying. Recorded time was coded as wear time, non-wear time, or working time, based on diary recordings. Sleep and non-wear time were excluded. For a day to be considered valid the following rules were applied: ≥10 h of worn waking hours, < 95% of time spent in any one behaviour (sedentary, standing, walking), and ≥ 500 steps [22]. Work time was considered valid when the device was worn for ≥80% of the time at work and ≥ 5 h of worn working hours. Data from at least 4 days were required, with at least 2 working days and 2 non-working days. The primary outcome extracted from this device was total sedentary time (% of wear time), averaged over all days. Secondary outcome measures (% of wear time) were: standing and walking, averaged over all days; and sedentary, standing, and walking for work time only.

##### *Self-reported physical activity and sedentary behaviour (secondary outcomes)*

Two validated questions for physical activity were used, one regarding exercise and one regarding daily activities (see supplementary file 1) [23]. Self-reported physical activity was based on an index of these two questions as described previously [23]. The index was then dichotomised into more favourable vs. more unfavourable physical activity levels using a cut-off coinciding with approximately 150 min/week of accelerometer assessed MVPA [23].

For sedentary behaviour, a validated question accounting for the amount of sitting time, excluding sleep, was used (see supplementary file 1) [24]. There were 7 possible response options. A cut-off of less than 7–9 h was used to classify participants as more favourable or more unfavourable sedentary behaviour. This cut-off has shown correspondence to approximately 10 h of accelerometer assessed sedentary time [24].

### Statistical analyses

The power calculation are presented in detail in the published protocol [18].

### Group comparisons

We planned to perform multilevel regression models (frequentist analyses) to evaluate differences in effects between the iPA, iSED, and control group (C) [18]. However, when running these models issues arose regarding the singular fit. A singularity error appeared in about 25% of the models, but with no clear pattern of occurrence. We chose to perform multilevel models using Bayesian statistics for all outcomes instead, because it is a preferred method that can handle issues of singularity [25]. The frequentist multilevel results (β, and 95% confidence interval (CI) were presented for two main outcome variables (%MVPA and %Sedentary) in the complete cases dataset, to confirm the Bayesian statistics. Note that these two models did not have a singularity error.

Bayesian regression estimates and 95% credible intervals were estimated using the packages with R statistical program language in Rstudio (R version 3.6.1, packages: brms, tidyverse and tidybayes). Bayesian analysis has a number of advantages, e.g. it can be applied to a large range of models including hierarchical multilevel models, the calculation of exact estimates without reliance on large sample sizes, and provides comprehensible answers with credible intervals that can be interpreted as probabilities [25].

### Models

In all models, clustering of data was taken into account using a two-level structure with participants as the second level and clusters as the first level. All groups were simultaneously regressed on the post-test value of the outcome, adjusted for the baseline value of the outcome with age, gender, education, and company as covariates. We used an uninformative prior (student t) for the coefficients. For all device-measured physical activity and sedentary behaviour, which were continuous outcomes, the Gaussian function was used (4 chains, 4 cores and 3000 iterations, 1000 warm-up). Parameter estimates and 95% credible intervals were presented. For the analyses on the dichotomised self-reported physical activity and sedentary behaviour outcome variables, the bernoulli function was used (4 chains, 4 cores and 3000 iterations, 1000 warm-up). For these outcomes, parameter estimates and credible intervals were exponentiated to provide posterior odds ratios. The achieved level of convergence was Rhat values of 1 for all models.

Furthermore, we determined posterior probabilities, which is the probability that the coefficient for a group comparison is above 0. Being above (or below) 0 with a high probability indicates that there was a difference between groups in the outcome variable. Although Bayesian models should not be interpreted like frequentist models regarding significance, we used a cut-off for indicating significant effects of these posterior probabilities as below 0.025 or above 0.975, which is comparable to the traditional *p*-value significance level of 0.05.

### Datasets

For each of the different defined outcome variables, separate models were run and repeated for each of the following four datasets:

A. Complete cases. The analyses performed with this dataset were considered as the primary results. Education was imputed with median values for 7 participants who had missings or impossible values but no further imputations were performed. There were no missing values for gender or age.
B. Intention To Treat with last observation carried forward (ITT-LOCF). The assumption in this model was that individuals with missing values at follow-up have no change from baseline. The imputation was performed in 3 steps:

i. Missing values for education were imputed using k Nearest Neighbour imputation using baseline variables.
ii. Missing values for the outcomes at baseline were imputed with follow-up values if available, otherwise with k Nearest Neighbour imputation.
iii. Missing values at follow-up were imputed with Last Observation Carried forward, which in this case is the baseline observation carried forward.

C. Intention To Treat with k Nearest Neighbour (ITT-kNN). All missing values were imputed with k Nearest Neighbour imputation.
D. Per Protocol. The inclusion criteria for this dataset were:

i. Individuals in iPA or iSED groups who had attended 3 to 5 counselling sessions [18], or individuals in C group. For those individuals that did not have this information available, it was assumed that they did not participate in at least 3 sessions.
ii. Individuals with at least one non-missing value at follow-up, meaning that they should have at least results of either one of the device-measured or self-reported physical activity or sedentary behaviour outcomes at follow-up.

Education was imputed with median values for those who had missings or impossible values but no further imputations were performed.

### Drop-out analyses

Drop-outs were defined as having neither Actigraph nor ActivPAL data at follow-up. To determine whether missing data were not at random, we performed binary logistic regression analyses using generalised linear models. Differences between those who dropped-out and those who did not drop out were assessed for: group allocation, age, gender, education, as well as baseline physical activity (%MVPA, Actigraph) and baseline sedentary behaviour (%Sedentary, ActivPAL). These analyses were performed using SPSS (Statistical Package of Social Sciences, version 25).

## Results

### Participants

**Fig. 1:**
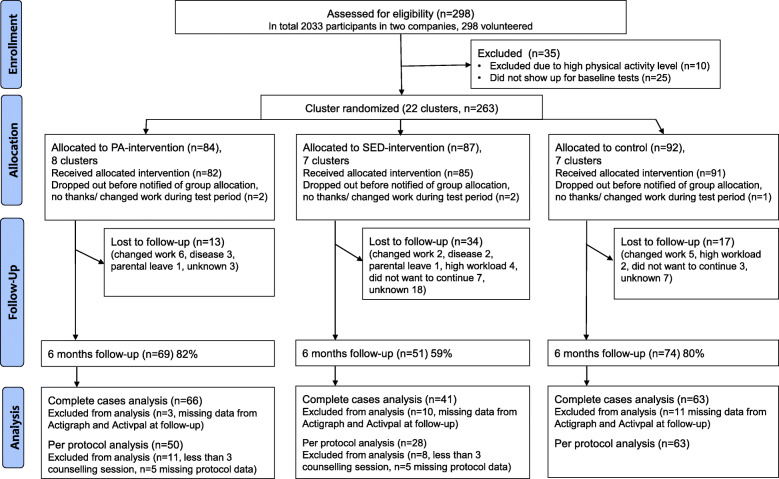
Flowchart of enrolment, participation, and analysis

A flowchart with the enrolment, participation, and analysis is presented in Fig. 1. A total of 263 office workers were randomised into 22 clusters at baseline. After 6 months, 194 (75%) participants remained, with the participation rate ranging from 59 to 82% between groups. Participants’ characteristics of each group are shown in Table 1. Observed data on baseline and post-test measurements, specified per allocated group are shown in Table 2. No harms were reported from the participants.

**Table 1:** Participants’ baseline characteristics

	iPA *n* = 84, 8 clusters	iSED *n* = 87, 7 clusters	C *n* = 92, 7 clusters
Age, mean (SD) years	41 (9)	41 (9)	44 (8)
Female, % (n)	80 (67)	64 (56)	76 (70)
Education, mean (SD) years	15 (2)	14 (2)	15 (2)
Married/living together, % (n)	78 (65)	85 (71)	79 (72)
Children at home under 18, % (n)	54 (45)	69 (59)	67 (61)
Working fulltime, % (n)	88 (73)	94 (80)	93 (85)
Current smoker, % (n)	11 (9)	7 (6)	1 (1)
BMI, mean (SD)	25 (4)	25 (4)	26 (5)

*iPA* Physical activity intervention, *iSED* Sedentary behaviour intervention, *C* Control group, *SD* Standard deviation, *BMI* Body mass index

**Table 2:** Observed data on baseline and post-test measurements, specified per allocated group

		iPA (8 clusters)		iSED (7 clusters)		C (7 clusters)	
		Baseline	Post-test	Baseline	Post-test	Baseline	Post-test
Physical activity Actigraph, mean (SD)		*n* = 83	*n* = 65	*n* = 85	*n* = 41	*n* = 91	*n* = 61
% Average of all days	MVPA	6·3 (2·3)	6·2 (2·2)	6·4 (2·3)	6·6 (2·3)	6·3 (2·2)	5·9 (2·0)
	Light	33·3 (5·8)	32·4 (6·0)	32·1 (6·2)	33·1 (6·1)	32·5 (6·1)	31·6 (6·4)
	Moderate	5·4 (1·8)	5·3 (1·8)	5·5 (1·9)	5·7 (2·2)	5·4 (1·7)	5·2 (1·7)
	Vigorous	0·9 (1·0)	0·8 (0·9)	0·8 (0·9)	0·7 (0·7)	0·8 (0·7)	0·7 (0·6)
% Average of weekdays only	MVPA	6·0 (2·3)	6·1 (2·2)	6·1 (2·4)	6·1 (2·1)	6·0 (2·1)	5·8 (1·9)
	Light	30·9 (5·6)	30·1 (6·3)	29·7 (5·9)	30·8 (5·8)	29·9 (6·2)	29·4 (6·8)
	Moderate	5·1 (1·7)	5·3 (1·8)	5·3 (1·9)	5·4 (1·9)	5·1 (1·7)	5·1 (1·6)
	Vigorous	0·8 (1·1)	0·7 (0·9)	0·8 (0·9)	0·6 (0·6)	0·7 (0·7)	0·6 (0·7)
Sedentary behaviour Activpal, mean (SD)		*n* = 80	*n* = 58	*n* = 85	*n* = 37	*n* = 89	*n* = 61
% Average of all days	Sedentary	61·0 (7·4)	61·3 (6·5)	62·6 (7·9)	60·4 (6·7)	59·2 (9·0)	60·4 (8·9)
	Standing	26·5 (6·7)	26·7 (5·7)	25·3 (6·5)	27·0 (6·3)	28·2 (7·6)	27·5 (7·8)
	Walking	12·6 (2·7)	12·0 (2·6)	12·1 (2·8)	12·6 (2·6)	12·6 (2·6)	12·1 (2·8)
% Average of work time only	Sedentary	66·9 (12·6)	67·1 (10·3)	67·9 (10·7)	65·4 (10·1)	64·9 (13·2)	66·9 (16·1)
	Standing	24·3 (11·7)	23·4 (9·9)	23·1 (9·8)	25·1 (9·4)	26·1 (11·9)	24·5 (14·9)
	Walking	8·8 (2·9)	9·4 (2·8)	8·9 (2·9)	9·5 (2·9)	9·0 (3·0)	8·7 (2·8)
Self-reported % more favourable		*n* = 82	*n* = 59	*n* = 85	*n* = 41	*n* = 89	*n* = 62
Physical activity		59	73	62	78	63	69
Daily sitting time		16	27	17	39	15	23

*iPA* Physical activity intervention, *iSED* Sedentary behaviour intervention, *C* Control group, *SD* Standard deviation. A more favourable self-reported physical activity was defined using a cut-off coinciding with approximately 150 min/week of accelerometer assessed MVPA. A more favourable self-reported daily sitting time was defined as less than 7–9 h

### Group comparisons

**Fig. 2:**
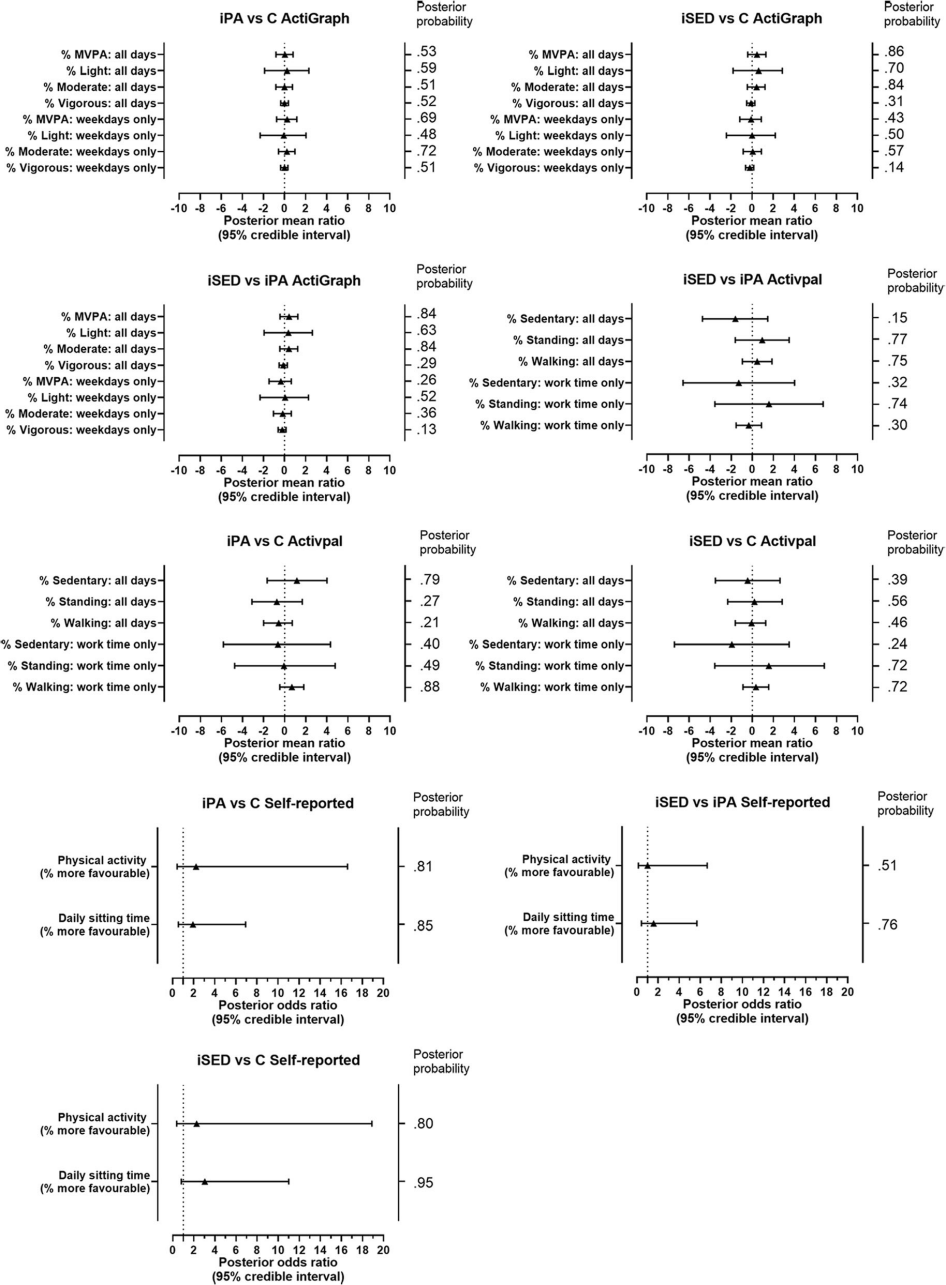
Forest plot of group differences for the complete cases dataset. iPA = physical activity intervention, iSED = sedentary behaviour intervention, C = control group, MVPA = moderate-to-vigorous physical activity. Posterior probability cut-off to indicate significant differences: > 0·975 or < 0·025

Figure 2 shows a forest plot of the Bayesian primary group comparison results for the complete cases data. Exact numbers can be found in supplementary file 2. No significant between-group differences were found for any of the physical activity or sedentary behaviour outcomes.

The group comparisons for the ITT and per protocol analyses can be found in supplementary file 2. Few significant differences were found in the ITT-kNN dataset when analysing Actigraph data, mostly reflecting more physical activity in the iSED group compared to the iPA group. No statistically significant differences were seen between the groups in the ITT-LOFC and the per protocol analyses.

Frequentist multilevel mixed models confirmed that there were no significant differences between groups for %MVPA or for %Sedentary. %MVPA: iPA vs C (β = 0·21, 95%CI = -0·55–0·97); iSED vs C (β = 0·63, 95%CI = -0·20–1·46); iSED vs iPA (β = 0·42, 95%CI = -0·39–1·22). %Sedentary: iPA vs C (β = 1·34, 95%CI = -1·22–3·90); iSED vs C (β = − 0·52, 95%CI = -3·31–2·27); iSED vs iPA (β = − 1·86, 95%CI = -4·57–0·86).

### Drop-out analyses

Those who dropped-out (*n* = 93) had significantly higher odds to be enroled in the iSED group compared to the C group, (OR = 2·43, 95% CI = 1·33–4·48), and lower odds to be the iPA group compared to the iSED group (OR = 0·24, 95% CI = 0·12–0·48). No significant differences were found between the iPA and C group (OR = 0·59, 95% CI = 0·30–1·17).

Baseline %MVPA and %Sedentary did not differ significantly between those who dropped-out and those who did not (respectively: OR = 1·02, 95% CI = 0·91–1·14 and 1·03, 95% CI = 0·99–1·06). Those who dropped-out had higher odds to be men (OR = 1·91, 95% CI = 1·09–3·34), lower odds to be older (OR = 0·97, 95% CI = 0·94–1·00), and lower odds to have a longer education (OR = 0·89, 95% CI = 0·80–0·99).

Of those who did not drop out, per protocol information on the amount of counselling sessions attended were missing for 10 participants.

## Discussion

This cluster RCT amongst office workers was the first to compare two multi-component interventions, targeting either physical activity or sedentary behaviour, with a control group. Results indicate that both multi-component interventions were unsuccessful at increasing physical activity and reducing sedentary time. Comparing the two interventions also indicated that none of them was superior to the other. Findings were robust for both device-measured and self-reported physical activity and sedentary behaviour. Furthermore, the lack of effect was also consistent regardless of analysing complete cases, intention to treat, or per protocol datasets. Although interventions were theory-based and office workers and companies’ preferences were taken into account when designing the interventions, in practice, these interventions were not able to change the office workers’ physical activity or sedentary behaviour as measured directly after the 6-month interventions. As many previous studies have encountered, changing behaviour is a major challenge [26].

### Reflections on the lack of effect

One explanation for the lack of effect might be the relatively favourable baseline levels of MVPA in the participants compared to the total population [2]. Although we specifically aimed at targeting an inactive population and we excluded those that were very active, many of the office workers were already relatively physically active at baseline. However, baseline device-measured behaviour might also have been positively biased. Even though device-measured outcomes are considered more objective compared to self-reported outcomes, participants might be influenced by the awareness of wearing these devices [27]. In an intervention study, this effect may be even stronger since the prospect of participating in a behavioural change study in itself might already change behaviour. This hypothesis is supported by our descriptive data that indicated that the control group consistently had more favourable physical activity and sedentary behaviour at baseline compared to follow up, despite the fact that measurements were spread over seasons. As a result of the potential bias towards more favourable device-measured physical activity patterns at baseline, part of the effects might have been masked. There is however no reason to believe that this effect has been larger in our trial compared to previous trials that did find significant effects of interventions on similar outcomes [8, 9]. It does stress the importance of including a control group in trials with a similar design. Another explanation for the lack of effect might be that participants felt that the intervention with five counselling sessions was too time consuming and ambitious. Future studies should work towards finding a better balance between what is needed based on theory and what is well-received by the target group.

A surprising finding was that more people dropped out from the sedentary behaviour intervention compared to the physical activity intervention. As participants were randomised into either of these groups after baseline it might indicate that the intervention targeting sedentary behaviour was less attractive compared to the physical activity intervention. One factor that might have influenced the attractiveness was that only participants in the physical activity intervention received free access to a gym. Another consideration is that even though the awareness of sedentary behaviour and potential health effects seem to be growing, the belief that this behaviour needs to be changed might still not be as accepted compared to changing physical inactivity [28]. Further research should therefore consider these aspects.

### Comparison with other studies

Several other intervention studies aiming at improving physical activity amongst office workers also did not manage to successfully change this behaviour. A study amongst employees with mixed professions who participated in a similar counselling intervention on physical activity, with one group only receiving counselling and another group receiving counselling and fitness testing, also showed no effects on physical activity compared to a control group [29]. A recent study on a 6-month workplace intervention with a different approach, studying a loyalty scheme with financial incentives, also did not show a significant improvement in physical activity [30].

With regard to studies targeting sedentary behaviour, two recent large cluster RCTs amongst office workers did manage to reduce sitting time in multi-component interventions [31, 32]. Compared to the current study, all participants in these previous studies did not have a sit-stand desk at baseline but were provided one as part of the intervention. Therefore, the contrast from baseline to post-intervention was larger than in our study were the companies as a standard provide all their office workers with sit-stand desks and office workers were simply encouraged to use them more often. Furthermore, both of these previous trials had an organisational intervention component that involved senior management as compared to the present trial in which team leaders played the primary role in the organisational part. Although some of the team leaders in the current study were also team managers, in the majority of the clusters, team leaders did not have a management function. Involving senior management might thus be a key component for success, and further studies should consider this.

Different from previous studies [29–32], our aim was to increase physical activity or reduce sedentary behaviour in order to improve mental health and cognition [18]. Therefore, how the intervention was presented to participants may have altered their attitudes or behaviours. This is however outside the scope of the current paper. After this RCT, the study continued as a cohort study with the control group randomised into either of the interventions, and others remaining in their intervention groups. Planned studies on mental health and cognition, as well as on the working mechanisms and feasibility of the interventions will be published separately and will provide more insight and practical directions on how to change physical activity behaviour in order to improve mental health and cognition.

### Limitations

The original aim of this study was to include 330 persons, but unfortunately, this target was not reached, thus the study may be underpowered. It is a common challenge in intervention studies with healthy volunteers to recruit the desired number of participants. Additionally, the included participants might not be fully representative for all office workers. Thus, results have to be interpreted with caution. Based on spontaneous reports, we can only speculate on why persons did not volunteer to be part of the study and/or dropped out. Some reasons were high workload, high staff turnover, and no time for physical activity during worktime. Future studies might consider incorporating stratified inclusion of participants and incorporating more support components specifically requested from previously underrepresented groups.

As described earlier, the sample was relatively physically active at baseline, which might have further negatively affected the statistical power as less participants were able to improve their physical activity. On the other hand, the lack of effectiveness of interventions was a robust finding over all physical activity and sedentary behaviour outcomes and datasets.

The cluster randomised design of this study is a strength, but since office workers were randomised into teams spread over three offices we cannot rule out the risk of contamination between groups. Plus, variability in implementation could have occurred across clusters at the organisational and environmental levels, since administration was primarily performed via team leaders. Although this study had a considerable amount of drop-outs, the rate was only slightly higher compared to other similar trials amongst office workers [31, 32]. Furthermore, a large proportion of women participated in this study, and more men dropped out of the study, which limits the generalisability of the study. Also, younger persons and persons with longer education were more likely to drop out. This might be interpreted as the intervention being experienced as less attractive to men, younger, and higher educated office workers. Future studies should consider gender, age, and education more carefully in the design of interventions.

## Conclusions

The multi-component interventions, either targeting physical activity or sedentary behaviour, did not lead to an increase in physical activity or a reduction in sedentary behaviour amongst office workers. The sedentary behaviour intervention was as ineffective as the physical activity intervention. Findings were consistent over both device-measured and self-reported behaviour measures, and there was no effect when only analysing those that adhered to the intervention protocol. Future studies should develop more effective strategies to increase physical activity and reduce sedentary behaviour, and consider the attractiveness of these interventions with regard to gender, age, and education.

## Supplementary information

**Additional file 1.**

**Additional file 2.**

## Data Availability

The datasets generated and/or analysed during the current study are not publicly available due to that the original approval by the regional ethics board and the informed consent from the participants do not include such direct free access, but are available from the corresponding author on reasonable request.

## Abbreviations

CBT: Cognitive behavioural therapy
BMI: Body Mass Index
C: Control group
CI: Confidence interval
cpm: Counts per minute
HR: Human Resources
iPA: Intervention targeting physical activity
iSED: Intervention targeting sedentary behaviour
ITT-kNN: Intention To Treat with k Nearest Neighbour
ITT-LOCF: Intention To Treat with last observation carried forward
MVPA: Moderate-to-vigorous physical activity
OR: Odds ratio
RCT: Randomised controlled trial
SD: Standard deviation
VM: Vector magnitude
β: Beta coefficient
