# Supplementary material for: The effectiveness of multi-component interventions targeting physical activity or sedentary behaviour amongst office workers: a three-arm cluster randomised controlled trial
Source: BMC Public Health. 2020 Sep 1;20:1329. doi: 10.1186/s12889-020-09433-7 (PMC7466462; doi:10.1186/s12889-020-09433-7)
Supplement: Supplementary file 1 — Additional file 1. [file 12889_2020_9433_MOESM1_ESM.docx]

**Supplementary file 1.**

Self-reported physical activity

*Exercise*

During a regular week, how much time do you spend exercising on a level that makes you short winded, for example running, fitness class, or ball games?

1. 0 minutes
2. Less than 30 minutes
3. 30–59 minutes (0,5–1 hour)
4. 60–89 minutes (1–1,5 hours)
5. 90–120 minutes (1,5–2 hours)
6. More than 120 minutes (>2 hours)

*Everyday physical activity*

During a regular week, how much time are you physically active in ways that are not exercise, for example walks, bicycling, or gardening? Add together all activities lasting at least 10 minutes.

1. 0 minutes
2. Less than 30 minutes
3. 30–59 minutes (0,5–1 hour)
4. 60–89 minutes (1–1,5 hours)
5. 90–149 minutes (1,5–2,5 hours)
6. 150–300 minutes (2,5–5 hours)
7. More than 300 minutes (>5 hours)

***Total self-reported physical activity***

Total physical activity was calculated by combining the results on exercise and physical activity. To consider an assumed higher intensity of exercise compared to daily physical activity, the weight of exercise in the formula was doubled.(Olsson et al. 2016)
For example, for someone reporting “Less than 60-89 minutes minutes” of exercise (answer 4) and “90-149 minutes” of everyday physical activity (answer 5), total score was: 4*2 + 5 = 13.

The range of possible scores was thus 3 to 19. A cut-off of at least 11 was used to classify participants as having favourable physical activity levels, as this level has found to correspond with the physical guidelines of 150 min/week, as accessed with accelerometers. (Olsson et al. 2016)

Self-reported sedentary behaviour

How much time do you sit on a normal day, excluding sleep?

- Virtually all day
- 13-15 hours
- 10-12 hours
- 7-9 hours
- 4-6 hours
- 1-3 hours
- Never
