# Supplementary material for: The effectiveness of multi-component interventions targeting physical activity or sedentary behaviour amongst office workers: a three-arm cluster randomised controlled trial
Source: BMC Public Health. 2020 Sep 1;20:1329. doi: 10.1186/s12889-020-09433-7 (PMC7466462; doi:10.1186/s12889-020-09433-7)
Supplement: Supplementary file 2 — Additional file 2. [file 12889_2020_9433_MOESM2_ESM.docx]

**Supplementary file 2.**

Bayesian model group comparison results for datasets:
A. Complete cases
B. Intention To Treat (ITT), Last observation carried forward (LOCF)
C. IntentionTo Treat (ITT), k Nearest Neighbour (kNN)
D. Per Protocol

**A. Bayesian model group comparison results for the complete cases data set**

|  | | Complete cases  *iPA: n=66, iSED: n=41, C: n=63* | | | | |
| --- | --- | --- | --- | --- | --- | --- |
|  |  | iPA vs C | iSED vs C | | | iSED vs iPA |
|  |  | Estimate (95% Credible interval) | | | | |
| Physical activity ^a^ Actigraph | |  | | | | |
| %  Average of  all days | MVPA | 0·04 (-0·80 – 0·82) | | 0·47 (-0·41 – 1·32) | 0·43 (-0·42 – 1·27) | |
|  | Light | 0·24 (-1·89 – 2·32) | | 0·62 (-1·80 – 2·87) | 0·38 (-1·93 – 2·65) | |
|  | Moderate | 0·01 (-0·81– 0·76) | | 0·43 (-0·45 – 1·23) | 0·42 (-0·44 – 1·25) | |
|  | Vigorous | 0·01 (-0·35 – 0·37) | | -0·10 (-0·51 – 0·27) | -0·11 (-0·51 – 0·27) | |
| %  Average of  weekdays only | MVPA | 0·25 (-0·75 – 1·17) | | -0·09 (-1·16 – 0·88) | -0·34 (-1·46 – 0·65) | |
|  | Light | -0·06 (-2·30 – 2·04) | | 0·01 (-2·44– 2·21) | 0·07 (-2·32 – 2·28) | |
|  | Moderate | 0·23 (-0·56 – 0·99) | | 0·07 (-0·84 – 0·89) | -0·16 (-1·04 – 0·65) | |
|  | Vigorous | 0·00 (-0·36 – 0·33) | | -0·21 (-0·61 – 0·16) | -0·21 (-0·60 – 0·15) | |
| Sedentary behaviour ^a^ Activpal | |  | |  |  | |
| %  Average of  all days | Sedentary | 1·16 (-1·66 – 4·02) | | -0·44 (-3·50 – 2·64) | -1·60 (-4·72 – 1·47) | |
|  | Standing | -0·74 (-3·10 – 1·67) | | 0·21 (-2·33 – 2·84) | 0·95 (-1·61 – 3·51) | |
|  | Walking | -0·56 (-2·00 – 0·72) | | -0·07 (-1·61 – 1·28) | 0·49 (-0·95 – 1·86) | |
| %  Average of  work time only | Sedentary | -0·64 (-5·82 – 4·35) | | -1·94 (-7·39 – 3·50) | -1·31 (-6·55 – 4·03) | |
|  | Standing | -0·06 (-4·75 – 4·79) | | 1·58 (-3·55 – 6·84) | 1·64 (-3·57 – 6·71) | |
|  | Walking | 0·68 (-0·46 – 1·81) | | 0·35 (-0·87 – 1·56) | -0·33 (-1·53 – 0·88) | |
| Self-reported ^b^  (% more favourable) | | | |  |  | |
| Physical activity | | 2·23 (0·44 – 16·6) | | 2·27 (0·38 – 18·9) | 1·01 (0·14 – 6·66) | |
| Daily sitting time | | 1·93 (0·55 – 6·93) | | 3·04 (0·81 – 11·0) | 1·58 (0·42 – 5·68) | |

*^a^=Posterior Mean ratios; ^b^=Posterior Odds Ratios
*Posterior probability > 0·975 or <0·025*

|  | | ITT-LOCF  *iPA: n=84, iSED: n=87, C: n=92* | | | | |
| --- | --- | --- | --- | --- | --- | --- |
|  |  | iPA vs C | iSED vs C | | | iSED vs iPA |
|  |  | Estimate (95% Credible interval) | | | | |
| Physical activity ^a^ Actigraph | |  | | | | |
| %  Average of  all days | MVPA | 0·03 (-0·52 – 0·55) | | 0·35 (-0·22 – 0·88) | 0·32 (-0·25 – 0·87) | |
|  | Light | -0·31 (-1·58 – 0·98) | | 0·81 (-0·49 – 2·14) | 1·12 (-0·20 – 2·46) | |
|  | Moderate | 0·00 (-0·46 – 0·47) | | 0·29 (-0·20 – 0·77) | 0·29 (-0·20 – 0·77) | |
|  | Vigorous | 0·04 (-0·14 – 0·22) | | 0·04 (-0·14 – 0·22) | -0·00 (-0·18 – 0·18) | |
| %  Average of  weekdays only | MVPA | 0·15 (-0·51 – 0·79) | | 0·16 (-0·53 – 0·82) | 0·01 (-0·65 – 0·64) | |
|  | Light | -0·80 (-2·13 – 0·60) | | 0·37 (-1·01 – 1·77) | 1·17 (-0·21 – 2·55) | |
|  | Moderate | 0·13 (-0·39 – 0·66) | | 0·20 (-0·35 – 0·71) | 0·06 (-0·46 – 0·59) | |
|  | Vigorous | 0·04 (-0·16 – 0·23) | | -0·03 (-0·22 – 0·17) | -0·07 (-0·25 – 0·13) | |
| Sedentary behaviour ^a^ Activpal | |  | |  |  | |
| %  Average of  all days | Sedentary | 1·07 (-0·73 – 2·83) | | -0·03 (-1·92 – 1·74) | -1·11 (-2·92 – 0·64) | |
|  | Standing | -0·86 (-2·21 – 0·49) | | -0·06 (-1·45 – 1·41) | 0·80 (-0·60 – 2·21) | |
|  | Walking | -0·22 (-0·93 – 0·48) | | 0·06 (-0·67 – 0·76) | 0·28 (-0·47 – 0·97) | |
| %  Average of  work time only | Sedentary | 0·23 (-2·57 – 2·97) | | -1·31 (-4·23 – 1·59) | -1·53 (-4·48 – 1·22) | |
|  | Standing | -0·70 (-3·23 – 1·89) | | 0·96 (-1·57 – 3·70) | 1·66 (-0·92 – 4·32) | |
|  | Walking | 0·38 (-0·42 – 1·20) | | 0·20 (-0·64 – 1·01) | -0·18 (-1·02– 0·65) | |
| Self-reported ^b^  (% more favourable) | | | |  |  | |
| Physical activity | | 1.25 (0.43 – 3.89) | | 1·43 (0·50 – 4·19) | 1·14 (0·38 – 3·41) | |
| Daily sitting time | | 1.43 (0-50 – 4.05) | | 2·21 (0·81 – 6·45) | 1·55 (0·58 – 4·45) | |

**B. Bayesian model group comparison results for the ITT-LOCF dataset**

*^a^=Posterior Mean ratios; ^b^=Posterior Odds Ratios
*Posterior probability > 0·975 or <0·025*

|  | | ITT-kNN  *iPA: n=84, iSED: n=87, C: n=92* | | | | |
| --- | --- | --- | --- | --- | --- | --- |
|  |  | iPA vs C | iSED vs C | | | iSED vs iPA |
|  |  | Estimate (95% Credible interval) | | | | |
| Physical activity ^a^ Actigraph | |  | | | | |
| %  Average of  all days | MVPA | -0·22 (-0·99 – 0·55) | | 0·61 (-0·23 – 1·38) | 0·83 (0·01 – 1·64)* | |
|  | Light | -0·48 (-1·90 – 0·96) | | 1·03 (-0·38 – 2·52) | 1·51 (0·03 – 3·02)* | |
|  | Moderate | -0·18 (-1·09 – 0·74) | | 0·70 (-0·20 – 1·59) | 0·89 (-0·02 –1·73)* | |
|  | Vigorous | 0·04 (-0·19 – 0·25) | | -0·09 (-0·32 – 0·13) | -0·13 (-0·35 – 0·10) | |
| %  Average of  weekdays only | MVPA | -0·00 (-0·83 – 0·81) | | 0·57 (-0·30 – 1·35) | 0·57 (-0·25 – 1·38) | |
|  | Light | -0·46 (-2·12 – 1·16) | | 1·71 (0·12 – 3·37)* | 2·18 (0·55 – 3·84)* | |
|  | Moderate | -0·04 (-0·82 – 0·72) | | 0·52 (-0·30 – 1·29) | 0·57 (-0·24 – 1·32) | |
|  | Vigorous | 0·01 (-0·24 – 0·23) | | -0·17 (-0·43 – 0·05) | -0·18 (-0·41 – 0·05) | |
| Sedentary behaviour ^a^ Activpal | |  | |  |  | |
| %  Average of  all days | Sedentary | -0·00 (-2·14 – 2·10) | | -2·08 (-4·26 – 0·07) | -2·07 (-4·19 – 0·05) | |
|  | Standing | -0·01 (-1·85 – 1·92) | | 1·21 (-0·65 – 3·12) | 1·21 (-0·75 – 3·11) | |
|  | Walking | 0·20 (-0·43 – 0·87) | | 0·39 (-0·26 – 1·07) | 0·19 (-0·49 – 0·83) | |
| %  Average of  work time only | Sedentary | -1·38 (-5·08 – 2·18) | | -1·52 (-5·46 – 2·06) | -0·14 (-3·82 – 3·52) | |
|  | Standing | 1·06 (-2·65 – 4·88) | | 0·95 (-2·83 – 5·19) | -0·11 (-3·89 – 3·82) | |
|  | Walking | 0·61 (-0·17 – 1·38) | | 0·34 (-0·42 – 1·12) | -0·27 (-1·10 – 0·51) | |
| Self-reported ^b^  (% more favourable) | | | |  |  | |
| Physical activity | | 0·62 (0·24 – 1·64) | | 1·16 (0·42 – 3·34) | 1·87 (0·69 – 5·15) | |
| Daily sitting time | | 0·82 (0·29 – 2·26) | | 0·93 (0·32 – 2·55) | 1·14 (0·41 – 3·31) | |

**C. Bayesian model group comparison results for the ITT-kNN dataset**

*^a^=Posterior Mean ratios; ^b^=Posterior Odds Ratios
*Posterior probability > 0·975 or <0·025*

|  | | Per protocol  *iPA: n=50, iSED: n=28, C: n=63* | | | | |
| --- | --- | --- | --- | --- | --- | --- |
|  |  | iPA vs C | iSED vs C | | | iSED vs iPA |
|  |  | Estimate (95% Credible interval) | | | | |
| Physical activity ^a^ Actigraph | |  | | | | |
| %  Average of  all days | MVPA | 0·17 (-0·76 – 1·08) | | 0·67 (-0·43 – 1·72) | 0·50 (-0·54 – 1·54) | |
|  | Light | 0·05 (-2·26 – 2·26) | | 0·15 (-2·64 – 2·71) | 0·10 (-2·58 – 2·67) | |
|  | Moderate | 0·05 (-0·88 – 0·95) | | 0·72 (-0·33 – 1·76) | 0·67 (-0·35 – 1·70) | |
|  | Vigorous | 0·08 (-0·29 – 0·44) | | -0·11 (-0·57 – 0·30) | -0·19 (-0·63 – 0·23) | |
| %  Average of  weekdays only | MVPA | 0·49 (-0·51 – 1·48) | | 0·23 (-1·07 – 1·37) | -0·27 (-1·56 – 0·87) | |
|  | Light | -0·23 (-2·49 – 2·01) | | -0·08 (-2·93 – 2·48) | 0·14 (-2·69 – 2·74) | |
|  | Moderate | 0·37 (-0·48 – 1·24) | | 0·37 (-0·72 – 1·35) | 0·00 (-1·08 – 0·96) | |
|  | Vigorous | 0·07 (-0·32 – 0·43) | | -0·20 (-0·67 – 0·23) | -0·26 (-0·74 – 0·18) | |
| Sedentary behaviour ^a^ Activpal | |  | |  |  | |
| %  Average of  all days | Sedentary | 1·75 (-1·20 – 4·63) | | -0·64 (-4·31 – 2·92) | -2·40 (-5·89 – 1·09) | |
|  | Standing | -1·15 (-3·75 – 1·55) | | 0·14 (-3·00 – 3·45) | 1·28 (-1·80 – 4·53) | |
|  | Walking | -0·59 (-1·94 – 0·63) | | 0·38 (-1·34 – 1·78) | 0·97 (-0·53 – 2·33) | |
| %  Average of  work time only | Sedentary | 0·03 (-5·78 – 5·48) | | -2·57 (-9·69 – 4·07) | -2·60 (-9·28 – 3·85) | |
|  | Standing | -0·46 (-5·92 – 5·15) | | 2·32 (-3·87 – 8·95) | 2·77 (-3·34 – 9·13) | |
|  | Walking | 0·56 (-0·68 – 1·81) | | 0·44 (-1·13 – 1·91) | -0·12 (-1·61 – 1·33) | |
| Self-reported ^b^  (% more favourable) | | | |  |  | |
| Physical activity | | 3·06 (0·57 – 29·8) | | 2·39 (0·26 – 26·9) | 0·78 (0·06 – 7·34) | |
| Daily sitting time | | 2·22 (0·43 – 14·6) | | 2·27 (0·23 – 14·7) | 1·02 (0·09 – 6·33) | |

**D. Bayesian model group comparison results for the Per Protocol dataset**

*^a^=Posterior Mean ratios; ^b^=Posterior Odds Ratios
*Posterior probability > 0·975 or <0·025*
